# Supplementary material for: Quantitative Stimulated Emission Depletion (STED) Microscopy with DNA-Fluorophore Labels
Source: ACS Nano. 2026 Feb 3;20(6):5288–96. doi: 10.1021/acsnano.5c21411 (PMC12918720; doi:10.1021/acsnano.5c21411)
Supplement: Supplementary file 1 [file nn5c21411_si_001.pdf]

Quantitative stimulated emission depletion (STED) microscopy with DNA-fluorophore labels

Laurell F. Kessler[1], Yunqing Li[1], Ashwin Balakrishnan[1], Mike Heilemann[1,#]

[1]Institute of Physical and Theoretical Chemistry, Goethe-University Frankfurt, Max-von-Laue-Strasse 7, 60438 Frankfurt, Germany

[#]Corresponding author, contact: heilemann@chemie.uni-frankfurt.de

SUPPLEMENTAL INFORMATION

Supplemental Figures S1 - S6

Supplemental Tables S1 - S13

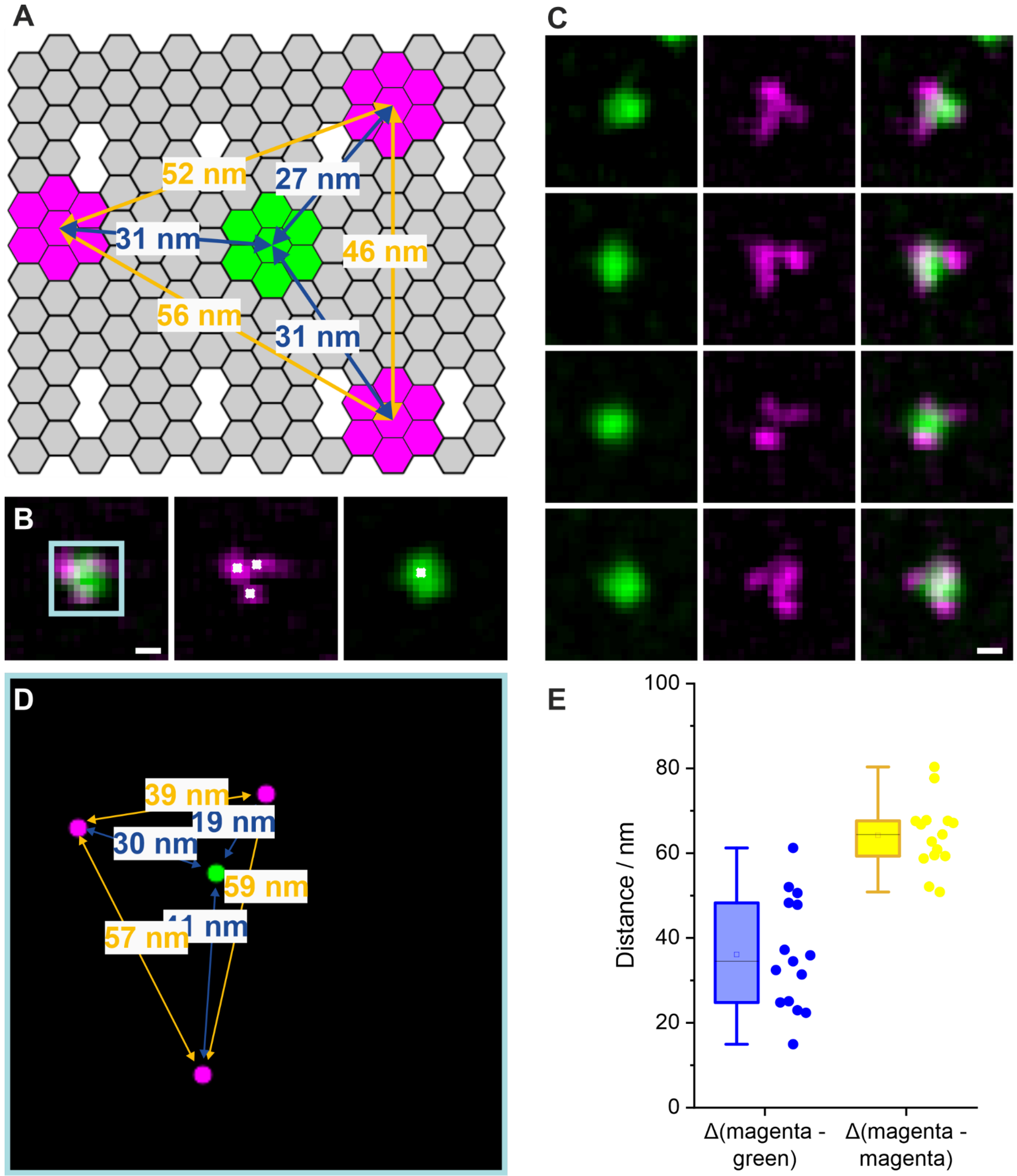

Figure S1: STED imaging of DNA origami and distance measurement. (A) Folding scheme of DNA origami and calculated distances assuming a hexagon size of 5 nm. (**B)** Exemplary two-color STED measurement of a single DNA origami (composite image, left; outside triangle, middle; center, right) with localized maxima indicated as white crosses. Scale bar = 50 nm. (**C)** Further examples of STED images of single DNA origami. Scale bar = 50 nm. (**D)** Zoom-in of the boxed region in (B) and inter-site distances. (**E)** Manual measured center-to-edge (blue) and edge-to-edge (yellow) distances from 5 DNA origami (B, C). Black lines indicate the median values, the squares indicate the mean

values, the box indicates the 25th and 75th percentiles, and the whiskers indicate the minimum and maximum.

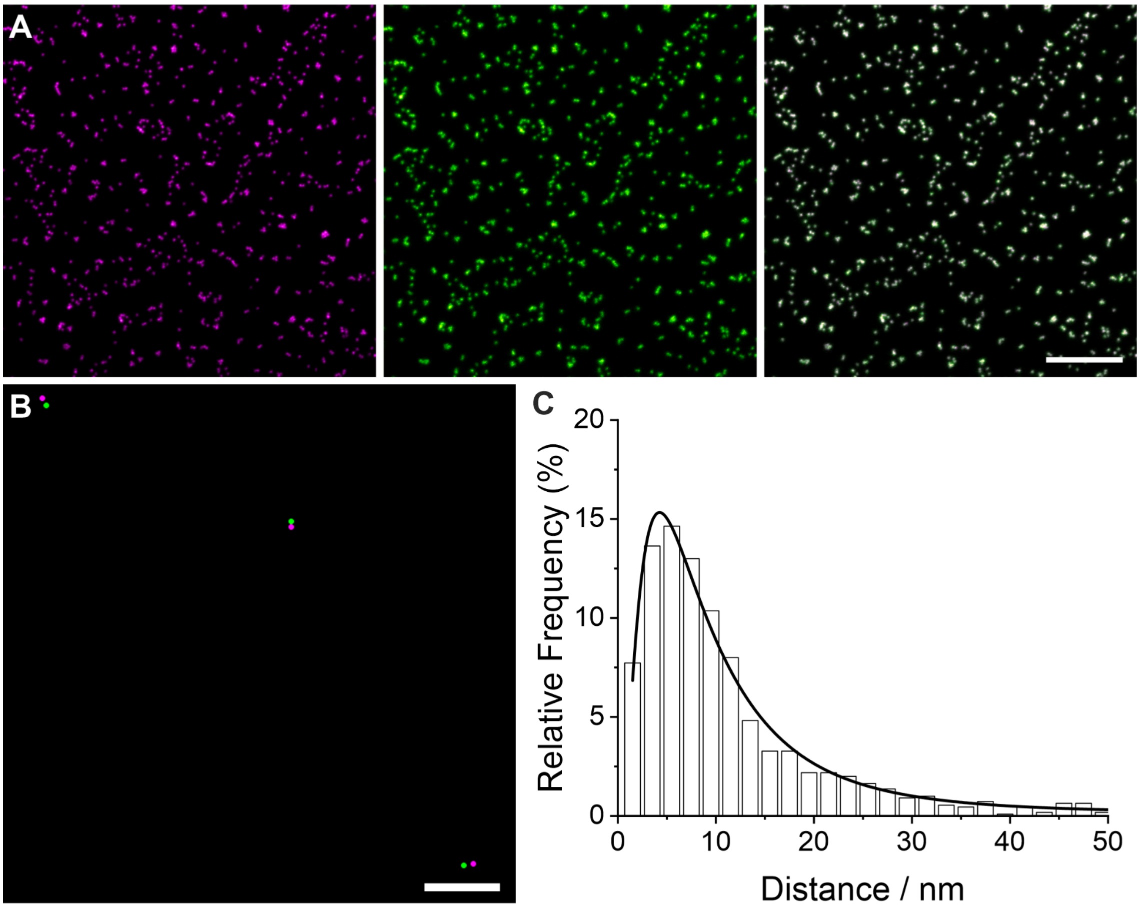

Figure S2: Chromatic aberration of the STED microscope assessed by measuring gold beads. (A) Two-color STED microscopy of gold beads on a glass surface using a 640 nm excitation laser (magenta; left) and a 561 nm excitation laser (green, middle). A composite of both channels is shown on the right. Scale bar = 3 µm. (**B)** Magnified view showing localized maxima of both channels. Scale bar = 50 nm. **C** Distribution of the nearest neighbor distance. A log-normal function was used to fit the histogram, with a peak at 4.0 +/- 0.1 nm.

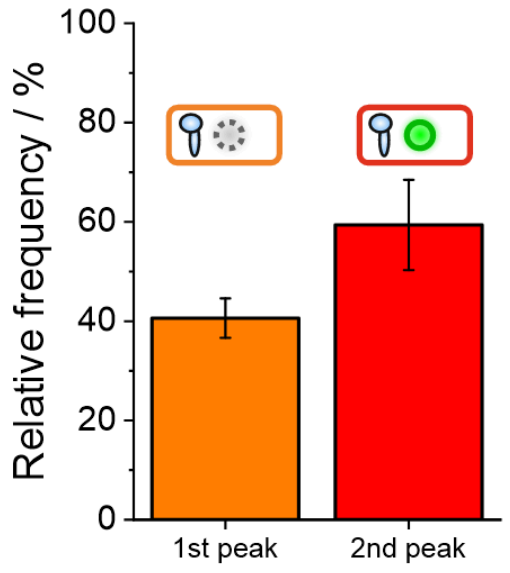

Figure S3: Detection efficiency of the ALFA-tag targeting nanobody. The 1st peak corresponds to the background signal, the 2nd peak corresponds to the fluorescence signal detected for NB@ALFA-4xP3. Relative occurrence was obtained by fitting two Gaussian distributions to the intensity distribution from resting cells (**Figure 4Ci**). From this, the correction factor for labeling was determined (equation (4), Methods).

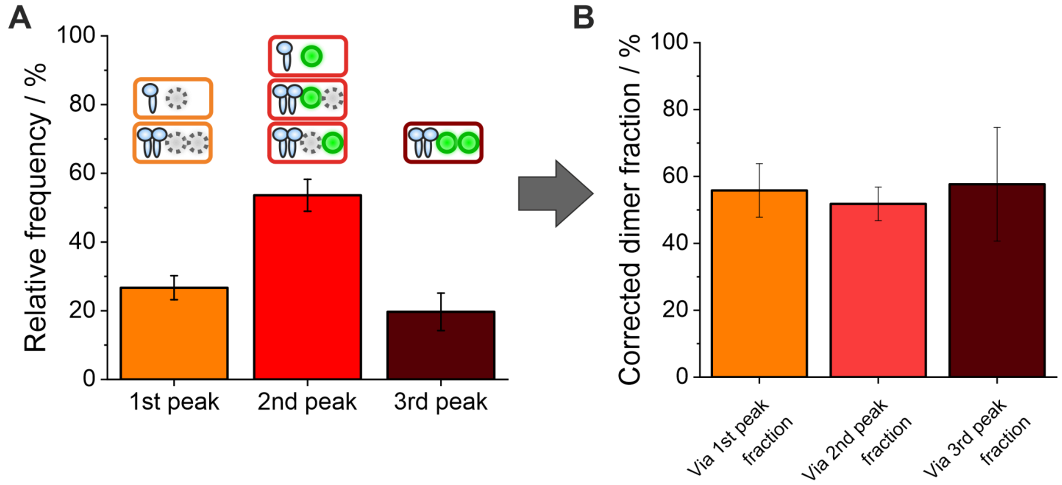

Figure S4: Absolute quantification of EGFR dimerization. (A) Normalized integrated intensity obtained by fitting 3 Gaussian distributions to the intensity histogram of EGF-stimulated cells (**Figure 4Cii**). The 1st peak corresponds to the background signal, the 2nd and 3rd peak are two intensity

levels detected for NB@ALFA-P3. **(B)** Fraction of EGFR dimerization corrected for NB@ALFA-P3 labeling efficiency from 1st (equation (5)), 2nd (equation (6)), and 3rd peak (equation (7)) (Methods).

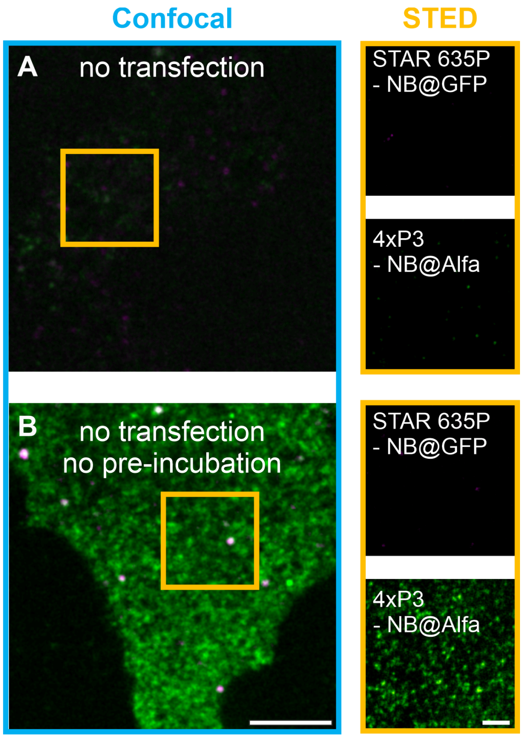

Figure S5: Negative controls using non-transfected CHO cells. (A) Exemplary untransfected cell measured with confocal and STED microscopy stained with NB@GFP-STAR 635P (magenta) and NB@ALFA-4xP3 (pre-incubated with P3-STAR 580; green). (**B)** Exemplary untransfected cell measured with confocal and STED microscopy stained with NB@GFP-STAR 635P (magenta) and NB@ALFA-4xP3 (no pre-incubation; green). Scale bar = 5 µm (confocal); 1 µm (STED). Images are displayed with identical dynamic range for each color channel and acquisition mode.

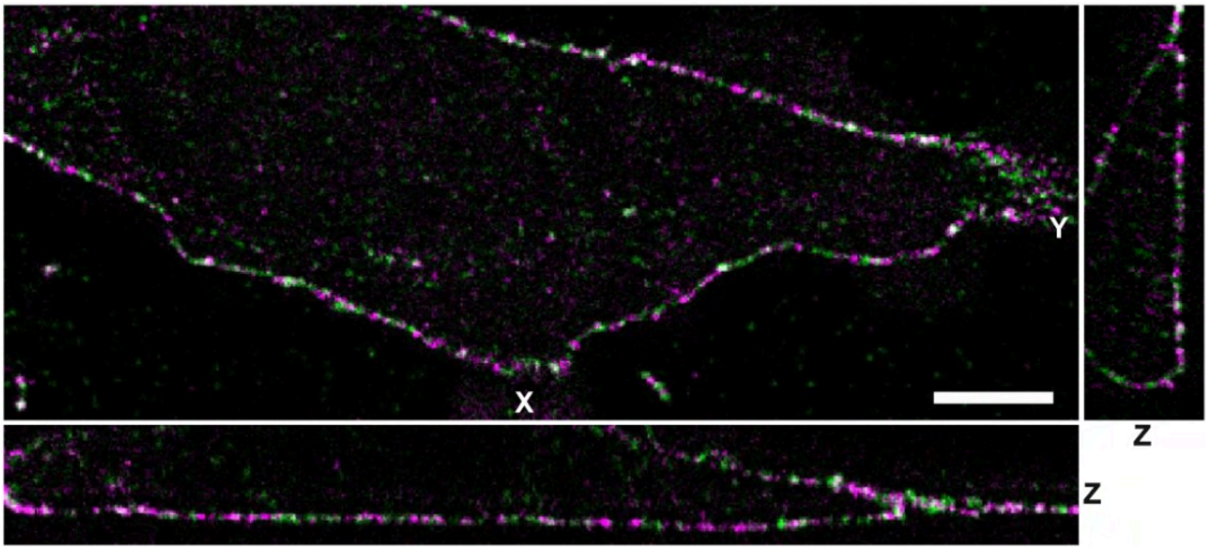

Figure S6: Volumetric STED microscopy of EGFR in a resting CHO cell. CHO cells expressing EGFR-ALFA-mEGFP were labeled with NB@ALFA-4xP3 (green) and NB@GFP-STAR 635P (magenta) (Scale bar = 4 µm).

Supplemental Tables

Table S1: Imager and docking strand sequences used in this study. Modified LNA nucleotides are colored blue (Guo et al. 2019). Docking sequences were synthesized together with the origami folding sequences from 5' to 3' end. Conjugation of the 4xP3 docking strand to the nanobody against GFP was performed by Massive Photonics (Germany).

**Name**	**Sequence**	**Supplier**
P2-STAR 635P	5' TATG**TAG**ATAA-STAR 635P-3'	biomers.net
P3-STAR 580	5' TAA**TGA**AGAAA -STAR 580-3'	biomers.net
2xP2 docking strand (Origami)	5' *Origami folding sequence* - TTATCTACATATTATCTACATA - 3'	Eurofins Genomics
4xP3 docking strand (Origami)	5' *Origami folding sequence* - TTTCTTCATTATTTCTTCATTATTTCTTCATTATTTCTTCATTA - 3'	Eurofins Genomics
4xP3 docking strand (Nanobody@GFP)	Nanobody - 5' - TTTCTTCATTATTTCTTCATTATTTCTTCATTATTTCTTCATTA - 3'	Massive Photonics

Table S2: Calculated distances from two-color STED measurements of DNA origamis.

**Pair**	**Distance / nm**
STAR 635P to STAR 580 (corner-to-center distance, magenta to green)	36 ± 14
STAR 635P to STAR 635P (corner-to-corner distance, magenta to magenta)	64 ± 8

Table S3: Intensity analysis of DNA origami with different numbers of target docking strands. Uncertainties are given as standard errors of the Gaussian fit.

	**1x**	**2x**	**3x**	**4x**	**5x**	**7x**
Mean / a. u.	8.9 ± 0.1	16.6 ± 0.1	21.1 ± 0.2	28.5 ± 0.2	35.4 ± 0.3	46.1 ± 0.2
FWHM / a. u.	11.2 ± 0.2	15.8 ± 0.3	18.1 ± 0.4	21.0 ± 0.4	24.9 ± 0.6	28.1 ± 0.7

Table S4: Fitting results for mixed DNA origami samples. Fixed values are indicated in red. Uncertainties are given as standard errors of the Gaussian fit.

	**1x+2x**		**1x+3x**		**1x+4x**		**1x+5x**		**1x+4x+7x**		
	Peak 1	Peak 2	Peak 1	Peak 2	Peak 1	Peak 2	Peak 1	Peak 2	Peak 1	Peak 2	Peak 3
Mean / a. u.	8.9	16.6	9.1 ± 0.2	21.9 ± 0.5	9.6 ± 0.1	30.0 ± 0.4	9.0 ± 0.2	37.2 ± 0.5	8.6 ± 0.3	27.3 ± 1.1	45.9 ± 3.6
FWHM / a. u.	11.2	15.8	11.2	18.1	11.2	21.0	11.2	24.9	11.2	21.0	28.1
Fraction / %	54 ± 1	46 ± 1	47 ± 2	53 ± 2	50 ± 1	50 ± 1	49 ± 2	51 ± 2	34 ± 1	44 ± 4	22 ± 4

Table S5: Alternative fitting models for the mixed samples of DNA origami containing 1x and 2x, or 1x and 4x and 7x, target docking strands. Fitting results with the lowest uncertainties are highlighted in blue.

	**1x+2x**				**1x+4x+7x**			
	2 peaks fitted (w1=w(1x)) (w2=w(2x))	2 peaks fitted (w1=w(1x)) (w2=w(3x))	1 peak fitted (w1=w(2x))	1 peak fitted (w1=w(1x))	3 peaks fitted (w1=w(1x)) (w2=w(4x)) (w3=w(7x))	3 peaks fitted (w1=w(1x)) (w2=w(3x)) (w3=w(7x))	2 peaks fitted (w1=w(1x)) (w2=w(5x))	2 peaks fitted (w1=w(1x)) (w2=w(7x))
Chi-squared	6E-6	2E-5	1E-5	6E-5	7E-6	8E-6	1E-5	1E-5
R-squared	0.995	0.986	0.986	0.957	0.981	0.980	0.958	0.965

Table S6: Fitting results for quantitative analysis of transfected EGFR in CHO cells. Fixed values are indicated in red. Uncertainties are given as the standard error of the Gaussian fit.

	**Resting cells**		**EGF-treated cells**		
	Peak 1 (background)	Peak 2 (signal monomer)	Peak 1 (background)	Peak 2 (signal monomer)	Peak 3 (signal dimer)
Mean / a. u.	2.2 ± 0.1	7.0 ± 0.8	2.7 ± 0.1	6.4 ± 0.9	16 ± 3
FWHM / a. u.	2.0 ± 0.1	11.0 ± 1.7	2.7 ± 0.3	11.0	15.5

Fraction / %	41 ± 4	59 ± 10	27 ± 4	54 ± 5	20 ± 6

Table S7: Corrected EGFR dimer fractions. Calculations were based on the assumption that transfected cells only exhibit EGFR monomers and dimers. Uncertainties are given as standard error.

	**Corrected dimer fraction / %**
First peak fraction	56 ± 8
Second peak fraction	52 ± 5
Third peak fraction	58 ± 17

Table S8: Cluster density of EGFR for transfected and untransfected cells. Uncertainties are reported as standard errors.

**Cell condition**	**Nanobody**	**Cluster density / µm^{-2}**
transfected resting	NB@GFP-STAR 635P	5.7 ± 1.3
	NB@ALFA-4xP3	6.3 ± 1.3
transfected EGF stimulated	NB@GFP-STAR 635P	5.4 ± 1.5
	NB@ALFA-4xP3	6 ± 3
no transfection	NB@GFP-STAR 635P	0.2 ± 0.1
	NB@ALFA-4xP3	0.4 ± 0.3
	NB@ALFA-4xP3 (no pre-incubation with imager strand)	10 ± 3

Table S9: Staple strand sequences and strand extensions (extension sequences are given in **Table S1**) of the rectangular DNA origami.

**Posi-tion**	**Sequence (5' to 3'; binding to scaffold)**	**Strand extension**
A1	TTTTCACTCAAAGGGCGAAAAACCATCACC	
B1	CAAATCAAGTTTTTTGGGGTCGAAACGTGGA	
C1	AGCTGATTGCCCTTCAGAGTCCACTATTAAAGGGTGCCGT	
D1	AAAGCACTAAATCGGAACCCTAATCCAGTT	
E1	AGCAAGCGTAGGGTTGAGTGTTGTAGGGAGCC	
F1	CCCGATTTAGAGCTTGACGGGGAAAAAGAATA	

G1	CCCAGCAGGCGAAAAATCCCTTATAAATCAAGCCGGCG	
H1	TCAATATCGAACCTCAAATATCAATTCCGAAA	
I1	AACGTGGCGAGAAAGGAAGGGAAACCAGTAA	
J1	TAAAAGGGACATTCTGGCCAACAAAGCATC	
K1	TCAACAGTTGAAAGGAGCAAATGAAAAATCTAGAGATAGA	
L1	ACCCTTCTGACCTGAAAGCGTAAGACGCTGAG	2xP2 docking strand (applied for all)
M1	CTTTAGGGCCTGCAACAGTGCCAATACGTG	2xP2 docking strand (applied for all)
N1	GCACAGACAATATTTTTGAATGGGGTCAGTA	2xP2 docking strand (applied for all)
O1	AGATTAGAGCCGTCAAAAAACAGAGGTGAGGCCTATTAGT	
P1	CTTTAATGCGCGAACTGATAGCCCCACCAG	
A2	GTCGACTTCGGCCAACGCGCGGGGTTTTTC	
B2	CTCCAACGCAGTGAGACGGGCAACCAGCTGCA	
D2	TGGAACAACCGCCTGGCCCTGAGGCCCGCT	
E2	CTGTGTGATTGCGTTGCGCTCACTAGAGTTGC	
F2	GCCCGAGAGTCCACGCTGGTTTGCAGCTAACT	
H2	GCAATTCACATATTCCTGATTATCAAAGTGTA	
I2	TCGGCAAATCCTGTTTGATGGTGGACCCTCAA	
J2	ACCTTGCTTGGTCAGTTGGCAAAGAGCGGA	
L2	AGCCAGCAATTGAGGAAGGTTATCATCATTTT	2xP2 docking strand (applied for all)
M2	CTACCATAGTTTGAGTAACATTTAAAATAT	2xP2 docking strand (applied for all)
N2	TTAACACCAGCACTAACAACTAATCGTTATTA	2xP2 docking strand (applied for all)
P2	CAGAAGATTAGATAATACATTTGTCGACAA	
A3	TGCATCTTTCCCAGTCACGACGGCCTGCAG	
B3	TTAATGAACTAGAGGATCCCCGGGGGGTAACG	
D3	TTCCAGTCGTAATCATGGTCATAAAAGGGG	
E3	GCTTTCCGATTACGCCAGCTGGCGGCTGTTTC	
F3	CACATTAAAATTGTTATCCGCTCATGCGGGCC	
H3	AGAAAACAAAGAAGATGATGAAACAGGCTGCG	
I3	AAGCCTGGTACGAGCCGGAAGCATAGATGATG	
J3	ATTATCATTCAATATAATCCTGACAATTAC	
L3	GCGGAACATCTGAATAATGGAAGGTACAAAAT	
M3	CATAAATCTTTGAATACCAAGTGTTAGAAC	2xP2 docking strand (applied for all)
N3	ATTTTAAAATCAAAATTATTTGCACGGATTCG	
P3	CTCGTATTAGAAATTGCGTAGATACAGTAC	
A4	TAATCAGCGGATTGACCGTAATCGTAACCG	
B4	CCAGGGTTGCCAGTTTGAGGGGACCCGTGGGA	
C4	GTATAAGCCAACCCGTCGGATTCTGACGACAGTATCGGCCGCAAGGCG	
D4	GATGTGCTTCAGGAAGATCGCACAATGTGA	
E4	ATATTTTGGCTTTCATCAACATTATCCAGCCA	
F4	TCTTCGCTGCACCGCTTCTGGTGCGGCCTTCC	
G4	TAAATCAAAATAATTCGCGTCTCGGAAACCAGGCAAAGGGAAGG	
H4	ATCGCAAGTATGTAAATGCTGATGATAGGAAC	
I4	CAACTGTTGCGCCATTCGCCATTCAAACATCA	
J4	CTGAGCAAAAATTAATTACATTTTGGGTTA	
K4	TCAAATATAACCTCCGGCTTAGGTAACAATTTCATTTGAAGGCGAATT	
L4	CGCGCAGATTACCTTTTTTAATGGGAGAGACT	
M4	CCTAAATCAAAATCATAGGTCTAAACAGTA	
N4	CCTGATTGCAATATATGTGAGTGATCAATAGT	
O4	GTGATAAAAAGACGCTGAGAAGAGATAACCTTGCTTCTGTTCGGGAGA	
P4	CTTTTACAAAATCGTCGCTATTAGCGATAG	
A5	AACGCAAAATCGATGAACGGTACCGGTTGA	
B5	ACAAACGGAAAAGCCCCAAAAACACTGGAGCA	
C5	TATATTTTGTCATTGCCTGAGAGTGGAAGATT	
D5	GCGAGTAAAAATATTTAAATTGTTACAAAG	
E5	TAGGTAAACTATTTTTGAGAGATCAAACGTTA	
F5	TGTAGCCATTAAAATTCGCATTAAATGCCGGA	
G5	GAGACAGCTAGCTGATAAATTAATTTTTGT	

H5	GTAATAAGTTAGGCAGAGGCATTTATGATATT	
I5	GCCATCAAGCTCATTTTTTAACCACAAATCCA	
J5	TATAACTAACAAAGAACGCGAGAACGCCAA	
K5	GTAAAGTAATCGCCATATTTAACAAAACTTTT	
L5	ACCTTTTTATTTTAGTTAATTTCATAGGGCTT	
M5	ACAACATGCCAACGCTCAACAGTCTTCTGA	
N5	GAATTTATTTAATGGTTTGAAATATTCTTACC	
O5	GTTTATCAATATGCGTTATACAAACCGACCGT	
P5	CTTAGATTTAAGGCGTTAAATAAAGCCTGT	
A6	AACAGTTTTGTACCAAAAACATTTTATTTC	2xP2 docking strand (applied for all)
B6	AACAAGAGGGATAAAAATTTTTAGCATAAAGC	
C6	GATTTAGTCAATAAAGCCTCAGAGAACCCTCA	
D6	GCTATCAGAAATGCAATGCCTGAATTAGCA	
E6	AATGGTCAACAGGCAAGGCAAAGAGTAATGTG	
F6	GAGGGTAGGATTCAAAAGGGTGAGACATCCAA	
G6	TTTGGGGATAGTAGTAGCATTAAAAGGCCG	
H6	CCAATAGCTCATCGTAGGAATCATGGCATCAA	4xP3 docking strand (applied for 7x)
I6	CAACCGTTTCAAATCACCATCAATTCGAGCCA	4xP3 docking strand (applied for 3x-5x,7x)
J6	CATGTAATAGAATATAAAGTACCAAGCCGT	4xP3 docking strand (applied for 3x-5x,7x)
K6	TATCCGGTCTCATCGAGAACAAGCGACAAAAG	
L6	AATTGAGAATTCTGTCCAGACGACTAAACCAA	
M6	GCGAACCTCCAAGAACGGGTATGACAATAA	
N6	AGTATAAAGTTCAGCTAATGCAGATGTCTTTC	
O6	GCCTTAAACCAATCAATAATCGGCACGCGCCT	
P6	TTAGTATCACAATAGATAAGTCCACGAGCA	
A7	TTTACCCCAACATGTTTTAAATTTCCATAT	2xP2 docking strand (applied for all)
B7	TAAATCGGGATTCCCAATTCTGCGATATAATG	2xP2 docking strand (applied for all)
C7	CGGATTGCAGAGCTTAATTGCTGAAACGAGTA	2xP2 docking strand (applied for all)
D7	AAATTAAGTTGACCATTAGATACTTTTGCG	
E7	CGAAAGACTTTGATAAGAGGTCATATTTCGCA	
F7	TAAATCATATAACCTGTTTAGCTAACCTTTAA	
G7	GCTTCAATCAGGATTAGAGAGTTATTTTCA	
H7	AGAGAGAAAAAAATGAAAATAGCAAGCAAACT	4xP3 docking strand (applied for 4x,5x,7x)
I7	TTCTACTACGCGAGCTGAAAAGGTTACCGCGC	4xP3 docking strand (applied for 1x-5x,7x)
J7	TTTTATTTAAGCAAATCAGATATTTTTTGT	4xP3 docking strand (applied for 7x)
K7	TTAGACGGCCAAATAAGAAACGATAGAAGGCT	
L7	GTACCGCAATTCTAAGAACGCGAGTATTATTT	
M7	AAAGTCACAAAATAAACAGCCAGCGTTTTA	
N7	CTTATCATTCCCGACTTGCGGGAGCCTAATTT	
O7	GAGAGATAGAGCGTCTTTCCAGAGGTTTTGAA	
P7	TGTAGAAATCAAGATTAGTTGCTCTTACCA	
A8	TTTAGGACAAATGCTTTAAACAATCAGGTC	2xP2 docking strand (applied for all)
B8	CTGTAGCTTGACTATTATAGTCAGTTCATTGA	2xP2 docking strand (applied for all)
C8	ATGCAGATACATAACGGGAATCGTCATAAATAAAGCAAAG	2xP2 docking strand (applied for all)
D8	GATGGCTTATCAAAAAGATTAAGAGCGTCC	
E8	TAAGAGCAAATGTTTAGACTGGATAGGAAGCC	
F8	TTGCTCCTTTCAAATATCGCGTTTGAGGGGGT	
G8	CGTTTACCAGACGACAAAGAAGTTTTGCCATAATTCGA	
H8	TTATTACGAAGAACTGGCATGATTGCGAGAGG	
I8	CCAACAGGAGCGAACCAGACCGGAGCCTTTAC	4xP3 docking strand (applied for 5x,7x)
J8	TTAACGTCTAACATAAAAACAGGTAACGGA	
K8	CGTAGAAAATACATACCGAGGAAACGCAATAAGAAGCGCA	
L8	ATCCCAATGAGAATTAACTGAACAGTTACCAG	
M8	AACGCAAAGATAGCCGAACAAACCCTGAAC	
N8	GCCAGTTAGAGGGTAATTGAGCGCTTTAAGAA	
O8	GTTTATTTTGTCACAATCTTACCGAAGCCCTTTAATATCA	

P8	ACGCTAACACCCACAAGAATTGAAAATAGC	
A9	CATCAAGTAAAACGAACTAACGAGTTGAGA	
B9	ATCCCCCTATACCACATTCAACTAGAAAAATC	
D9	AATACTGCCCAAAAGGAATTACGTGGCTCA	
E9	TCATTCAGATGCGATTTTAAGAACAGGCATAG	
F9	AATAGTAAACACTATCATAACCCTCATTGTGA	
H9	GCAAGGCCTCACCAGTAGCACCATGGGCTTGA	
I9	CTTTTGCAGATAAAAACCAAAATAAAGACTCC	
J9	ATACCCAACAGTATGTTAGCAAATTAGAGC	
L9	AAGGAAACATAAAGGTGGCAACATTATCACCG	
M9	TCAAGTTTCATTAAAGGTGAATATAAAAGA	
N9	AAGTAAGCAGACACCACGGAATAATATTGACG	
P9	AATAGCTATCAATAGAAAATTCAACATTCA	
A10	AATACGTTTGAAAGAGGACAGACTGACCTT	
B10	TACGTTAAAGTAATCTTGACAAGAACCGAACT	
D10	TTATACCACCAAATCAACGTAACGAACGAG	
E10	ACACTCATCCATGTTACTTAGCCGAAAGCTGC	
F10	ATTACCTTTGAATAAGGCTTGCCCAAATCCGC	
H10	TTGACAGGCCACCACCAGAGCCGCGATTTGTA	
I10	GATGGTTTGAACGAGTAGTAAATTTACCATTA	
J10	CAGCAAAAGGAAACGTCACCAATGAGCCGC	
L10	TCACCGACGCACCGTAATCAGTAGCAGAACCG	2xP2 docking strand (applied for all)
M10	TTAAAGCCAGAGCCGCCACCCTCGACAGAA	2xP2 docking strand (applied for all)
N10	GAAATTATTGCCTTTAGCGTCAGACCGGAACC	2xP2 docking strand (applied for all)
P10	ACCGATTGTCGGCATTTTCGGTCATAATCA	
A11	AGGCTCCAGAGGCTTTGAGGACACGGGTAA	
B11	GACCAACTAATGCCACTACGAAGGGGGTAGCA	
C11	TTTATCAGGACAGCATCGGAACGACACCAACCTAAAACGAGGTCAATC	
D11	GCGCAGACAAGAGGCAAAAGAATCCCTCAG	
E11	AAACAGCTTTTTGCGGGATCGTCAACACTAAA	
F11	GACCTGCTCTTTGACCCCCAGCGAGGGAGTTA	
G11	TGACAACTCGCTGAGGCTTGCATTATACCAAGCGCGATGATAAA	
H11	TTAGGATTGGCTGAGACTCCTCAATAACCGAT	
I11	TCATCGCCAACAAAGTACAACGGACGCCAGCA	
J11	CACCAGAAAGGTTGAGGCAGGTCATGAAAG	
K11	GCGGATAACCTATTATTCTGAAACAGACGATTGGCCTTGAAGAGCCAC	
L11	CCACCCTCTATTCACAAACAAATACCTGCCTA	2xP2 docking strand (applied for all)
M11	GTATAGCAAACAGTTAATGCCCAATCCTCA	2xP2 docking strand (applied for all)
N11	GCCTCCCTCAGAATGGAAAGCGCAGTAACAGT	2xP2 docking strand (applied for all)
O11	CAGGAGGTGGGGTCAGTGCCTTGAGTCTCTGAATTTACCGGGAACCAG	
P11	AAATCACCTTCCAGTAAGCGTCAGTAATAA	
A12	AGAAAGGAACAACTAAAGGAATTCAAAAAAA	
B12	ACGGCTACAAAAGGAGCCTTTAATGTGAGAAT	
C12	ACAACTTTCAACAGTTTCAGCGGATGTATCGG	
D12	CAGCGAAACTTGCTTTCGAGGTGTTGCTAA	
E12	TAAATGAATTTTCTGTATGGGATTAATTTCTT	
F12	AAGGCCGCTGATACCGATAGTTGCGACGTTAG	
G12	TCTAAAGTTTTGTCGTCTTTCCAGCCGACAA	
H12	TCCACAGACAGCCCTCATAGTTAGCGTAACGA	
I12	ATATTCGGAACCATCGCCCACGCAGAGAAGGA	
J12	TATTAAGAAGCGGGGTTTTGCTCGTAGCAT	
K12	TCACCAGTACAAACTACAACGCCTAGTACCAG	
L12	TTTCGGAAGTGCCGTCGAGAGGGTGAGTTTCG	
M12	AGGAACCCATGTACCGTAACACTTGATATAA	2xP2 docking strand (applied for all)
N12	GCCCGTATCCGGAATAGGTGTATCAGCCCAAT	
O12	CCACCCTCATTTTCAGGGATAGCAACCGTACT	
P12	GTTTTAACTTAGTACCGCCACCCAGAGCCA	

Table S10: Sequences of biotinylated staple strands for DNA origami.

Position	Sequence	Modification
C2	ATTAAGTTTACCGAGCTCGAATTCGGGAAACCTGTCGTGC	5'-biotin
C9	ATAAGGGAACCGGATATTCATTACGTCAGGACGTTGGGAA	5'-biotin
G2	GCGATCGGCAATTCCACACAACAGGTGCCTAATGAGTG	5'-biotin
G9	TTGTGTCGTGACGAGAAACACCAAATTTCAACTTTAAT	5'-biotin
K2	ATTCATTTTTGTTTGGATTATACTAAGAAACCACCAGAAG	5'-biotin
K9	CACCCTCAGAAACCATCGATAGCATTGAGCCATTTGGGAA	5'-biotin
O2	AACAATAACGTAAAACAGAAATAAAAATCCTTTGCCCGAA	5'-biotin
O9	AGCCACCACTGTAGCGCGTTTTCAAGGGAGGGAAGGTAAA	5'-biotin

Table S11: Sequence of scaffold strand (M13mp18) for DNA origami.

AATGCTACTACTATTAGTAGAATTGATGCCACCTTTTCAGCTCGCGCCCCAAATGAAAATATAGCTAAACAGGTTATTGACCATTTGCGAAATGTAT
CTAATGGTCAAACTAAATCTACTCGTTCGCAGAATTGGGAATCAACTGTTATATGGAATGAAACTTCCAGACACCGTACTTTAGTTGCATATTTAAA
ACATGTTGAGCTACAGCATTATATTCAGCAATTAAGCTCTAAGCCATCCGCAAAAATGACCTCTTATCAAAAGGAGCAATTAAAGGTACTCTCTAA
TCCTGACCTGTTGGAGTTTGCTTCCGGTCTGGTTCGCTTTGAAGCTCGAATTAAAACGCGATATTTGAAGTCTTTCGGGCTTCCTCTTAATCTTT
TTGATGCAATCCGCTTTGCTTCTGACTATAATAGTCAGGGTAAAGACCTGATTTTTGATTTATGGTCATTCTCGTTTTCTGAACTGTTTAAAGCATT
TGAGGGGGATTCAATGAATATTTATGACGATTCCGCAGTATTGGACGCTATCCAGTCTAAACATTTTACTATTACCCCCTCTGGCAAAACTTCTTT
TGCAAAAGCCTCTCGCTATTTTGGTTTTTATCGTCGTCTGGTAAACGAGGGTTATGATAGTGTTGCTCTTACTATGCCTCGTAATTCCTTTTGGCG
TTATGTATCTGCATTAGTTGAATGTGGTATTCCTAAATCTCAACTGATGAATCTTTCTACCTGTAATAATGTTGTTCCGTTAGTTCGTTTTATTAACG
TAGATTTTTCTTCCCAACGTCCTGACTGGTATAATGAGCCAGTTCTTAAAATCGCATAAGGTAATTCACAATGATTAAAGTTGAAATTAAACCATCT
CAAGCCCAATTTACTACTCGTTCTGGTGTTTCTCGTCAGGGCAAGCCTTATTCACTGAATGAGCAGCTTTGTTACGTTGATTTGGGTAATGAATA
TCCGGTTCTTGTCAAGATTACTCTTGATGAAGGTCAGCCAGCCTATGCGCCTGGTCTGTACACCGTTCATCTGTCCTCTTTCAAAGTTGGTCAG
TTCGGTTCCCTTATGATTGACCGTCTGCGCCTCGTTCCGGCTAAGTAACATGGAGCAGGTCGCGGATTTCGACACAATTTATCAGGCGATGATA
CAAATCTCCGTTGTACTTTGTTTCGCGCTTGGTATAATCGCTGGGGGTCAAAGATGAGTGTTTTAGTGTATTCTTTTGCCTCTTTCGTTTTAGGTT
GGTGCCTTCGTAGTGGCATTACGTATTTTACCCGTTTAATGGAAACTTCCTCATGAAAAAGTCTTTAGTCCTCAAAGCCTCTGTAGCCGTTGCTA
CCCTCGTTCCGATGCTGTCTTTCGCTGCTGAGGGTGACGATCCCGCAAAAGCGGCCTTTAACTCCCTGCAAGCCTCAGCGACCGAATATATCG
GTTATGCGTGGGCGATGGTTGTTGTCATTGTCGGCGCAACTATCGGTATCAAGCTGTTTAAGAAATTCACCTCGAAAGCAAGCTGATAAACCGA
TACAATTAAAGGCTCCTTTTGGAGCCTTTTTTTTGGAGATTTTCAACGTGAAAAAATTATTATTCGCAATTCCTTTAGTTGTTCCTTTCTATTCTCAC
TCCGCTGAAACTGTTGAAAGTTGTTTAGCAAAATCCCATACAGAAAATTCATTTACTAACGTCTGGAAAGACGACAAAACTTTAGATCGTTACGC
TAACTATGAGGGCTGTCTGTGGAATGCTACAGGCGTTGTAGTTTGTACTGGTGACGAAACTCAGTGTTACGGTACATGGGTTCCTATTGGGCTT
GCTATCCCTGAAAATGAGGGTGGTGGCTCTGAGGGTGGCGGTTCTGAGGGTGGCGGTTCTGAGGGTGGCGGTACTAAACCTCCTGAGTACG
GTGATACACCTATTCCGGGCTATACTTATATCAACCCTCTCGACGGCACTTATCCGCCTGGTACTGAGCAAAACCCCGCTAATCCTAATCCTTCT
CTTGAGGAGTCTCAGCCTCTTAATACTTTCATGTTTCAGAATAATAGGTTCCGAAATAGGCAGGGGGCATTAACTGTTTATACGGGCACTGTTAC
TCAAGGCACTGACCCCGTTAAAACTTATTACCAGTACACTCCTGTATCATCAAAAGCCATGTATGACGCTTACTGGAACGGTAAATTCAGAGACT
GCGCTTTCCATTCTGGCTTTAATGAGGATTTATTTGTTTGTGAATATCAAGGCCAATCGTCTGACCTGCCTCAACCTCCTGTCAATGCTGGCGGC
GGCTCTGGTGGTGGTTCTGGTGGCGGCTCTGAGGGTGGTGGCTCTGAGGGTGGCGGTTCTGAGGGTGGCGGCTCTGAGGGAGGCGGTTC
CGGTGGTGGCTCTGGTTCCGGTGATTTTGATTATGAAAAGATGGCAAACGCTAATAAGGGGGCTATGACCGAAAATGCCGATGAAAACGCGCT
ACAGTCTGACGCTAAAGGCAAACTTGATTCTGTCGCTACTGATTACGGTGCTGCTATCGATGGTTTCATTGGTGACGTTTCCGGCCTTGCTAAT
GGTAATGGTGCTACTGGTGATTTTGCTGGCTCTAATTCCCAAATGGCTCAAGTCGGTGACGGTGATAATTCACCTTTAATGAATAATTTCCGTCA
ATATTTACCTTCCCTCCCTCAATCGGTTGAATGTCGCCCTTTTGTCTTTGGCGCTGGTAAACCATATGAATTTTCTATTGATTGTGACAAAATAAAC
TTATTCCGTGGTGTCTTTGCGTTTCTTTTATATGTTGCCACCTTTATGTATGTATTTTCTACGTTTGCTAACATACTGCGTAATAAGGAGTCTTAATC
ATGCCAGTTCTTTTGGGTATTCCGTTATTATTGCGTTTCCTCGGTTTCCTTCTGGTAACTTTGTTCGGCTATCTGCTTACTTTTCTTAAAAAGGGC
TTCGGTAAGATAGCTATTGCTATTTCATTGTTTCTTGCTCTTATTATTGGGCTTAACTCAATTCTTGTGGGTTATCTCTCTGATATTAGCGCTCAATT
ACCCTCTGACTTTGTTCAGGGTGTTCAGTTAATTCTCCCGTCTAATGCGCTTCCCTGTTTTTATGTTATTCTCTCTGTAAAGGCTGCTATTTTCATT
TTTGACGTTAAACAAAAAATCGTTTCTTATTTGGATTGGGATAAATAATATGGCTGTTTATTTTGTAACTGGCAAATTAGGCTCTGGAAAGACGCTC
GTTAGCGTTGGTAAGATTCAGGATAAAATTGTAGCTGGGTGCAAAATAGCAACTAATCTTGATTTAAGGCTTCAAAACCTCCCGCAAGTCGGGA
GGTTCGCTAAAACGCCTCGCGTTCTTAGAATACCGGATAAGCCTTCTATATCTGATTTGCTTGCTATTGGGCGCGGTAATGATTCCTACGATGAA
AATAAAAACGGCTTGCTTGTTCTCGATGAGTGCGGTACTTGGTTTAATACCCGTTCTTGGAATGATAAGGAAAGACAGCCGATTATTGATTGGTT
TCTACATGCTCGTAAATTAGGATGGGATATTATTTTTCTTGTTCAGGACTTATCTATTGTTGATAAACAGGCGCGTTCTGCATTAGCTGAACATGTT
GTTTATTGTCGTCGTCTGGACAGAATTACTTTACCTTTTGTCGGTACTTTATATTCTCTTATTACTGGCTCGAAAATGCCTCTGCCTAAATTACATG
TTGGCGTTGTTAAATATGGCGATTCTCAATTAAGCCCTACTGTTGAGCGTTGGCTTTATACTGGTAAGAATTTGTATAACGCATATGATACTAAACA
GGCTTTTTCTAGTAATTATGATTCCGGTGTTTATTCTTATTTAACGCCTTATTTATCACACGGTCGGTATTTCAAACCATTAAATTTAGGTCAGAAGA
TGAAATTAACTAAAATATATTTGAAAAAGTTTTCTCGCGTTCTTTGTCTTGCGATTGGATTTGCATCAGCATTTACATATAGTTATATAACCCAACCT
AAGCCGGAGGTTAAAAAGGTAGTCTCTCAGACCTATGATTTTGATAAATTCACTATTGACTCTTCTCAGCGTCTTAATCTAAGCTATCGCTATGTTT
TCAAGGATTCTAAGGGAAAATTAATTAATAGCGACGATTTACAGAAGCAAGGTTATTCACTCACATATATTGATTTATGTACTGTTTCCATTAAAAAA
GGTAATTCAAATGAAATTGTTAAATGTAATTAATTTTGTTTTCTTGATGTTTGTTTCATCATCTTCTTTTGCTCAGGTAATTGAAATGAATAATTCGCC
TCTGCGCGATTTTGTAACTTGGTATTCAAAGCAATCAGGCGAATCCGTTATTGTTTCTCCCGATGTAAAAGGTACTGTTACTGTATATTCATCTGA
CGTTAAACCTGAAAATCTACGCAATTTCTTTATTTCTGTTTTACGTGCAAATAATTTTGATATGGTAGGTTCTAACCCTTCCATTATTCAGAAGTATA
ATCCAAACAATCAGGATTATATTGATGAATTGCCATCATCTGATAATCAGGAATATGATGATAATTCCGCTCCTTCTGGTGGTTTCTTTGTTCCGCA
AAATGATAATGTTACTCAAACTTTTAAAATTAATAACGTTCGGGCAAAGGATTTAATACGAGTTGTCGAATTGTTTGTAAAGTCTAATACTTCTAAAT
CCTCAAATGTATTATCTATTGACGGCTCTAATCTATTAGTTGTTAGTGCTCCTAAAGATATTTTAGATAACCTTCCTCAATTCCTTTCAACTGTTGAT
TTGCCAACTGACCAGATATTGATTGAGGGTTTGATATTTGAGGTTCAGCAAGGTGATGCTTTAGATTTTTCATTTGCTGCTGGCTCTCAGCGTGG
CACTGTTGCAGGCGGTGTTAATACTGACCGCCTCACCTCTGTTTTATCTTCTGCTGGTGGTTCGTTCGGTATTTTTAATGGCGATGTTTTAGGGC
TATCAGTTCGCGCATTAAAGACTAATAGCCATTCAAAAATATTGTCTGTGCCACGTATTCTTACGCTTTCAGGTCAGAAGGGTTCTATCTCTGTTG
GCCAGAATGTCCCTTTTATTACTGGTCGTGTGACTGGTGAATCTGCCAATGTAAATAATCCATTTCAGACGATTGAGCGTCAAAATGTAGGTATTT
CCATGAGCGTTTTTCCTGTTGCAATGGCTGGCGGTAATATTGTTCTGGATATTACCAGCAAGGCCGATAGTTTGAGTTCTTCTACTCAGGCAAGT
GATGTTATTACTAATCAAAGAAGTATTGCTACAACGGTTAATTTGCGTGATGGACAGACTCTTTTACTCGGTGGCCTCACTGATTATAAAAACACT

TCTCAGGATTCTGGCGTACCGTTCCTGTCTAAAATCCCTTTAATCGGCCTCCTGTTTAGCTCCCGCTCTGATTCTAACGAGGAAAGCACGTTATA
CGTGCTCGTCAAAGCAACCATAGTACGCGCCCTGTAGCGGCGCATTAAGCGCGGCGGGTGTGGTGGTTACGCGCAGCGTGACCGCTACACT
TGCCAGCGCCCTAGCGCCCGCTCCTTTCGCTTTCTTCCCTTCCTTTCTCGCCACGTTCGCCGGCTTTCCCCGTCAAGCTCTAAATCGGGGGC
TCCCTTTAGGGTTCCGATTTAGTGCTTTACGGCACCTCGACCCCAAAAAACTTGATTTGGGTGATGGTTCACGTAGTGGGCCATCGCCCTGATA
GACGGTTTTTCGCCCTTTGACGTTGGAGTCCACGTTCTTTAATAGTGGACTCTTGTTCCAAACTGGAACAACACTCAACCCTATCTCGGGCTAT
TCTTTTGATTTATAAGGGATTTTGCCGATTTCGGAACCACCATCAAACAGGATTTTCGCCTGCTGGGGCAAACCAGCGTGGACCGCTTGCTGCA
ACTCTCTCAGGGCCAGGCGGTGAAGGGCAATCAGCTGTTGCCCGTCTCACTGGTGAAAAGAAAAACCACCCTGGCGCCCAATACGCAAACC
GCCTCTCCCCGCGCGTTGGCCGATTCATTAATGCAGCTGGCACGACAGGTTTCCCGACTGGAAAGCGGGCAGTGAGCGCAACGCAATTAATG
TGAGTTAGCTCACTCATTAGGCACCCCAGGCTTTACACTTTATGCTTCCGGCTCGTATGTTGTGTGGAATTGTGAGCGGATAACAATTTCACACA
GGAAACAGCTATGACCATGATTACGAATTCGAGCTCGGTACCCGGGGATCCTCTAGAGTCGACCTGCAGGCATGCAAGCTTGGCACTGGCCG
TCGTTTTACAACGTCGTGACTGGGAAAACCCTGGCGTTACCCAACTTAATCGCCTTGCAGCACATCCCCCTTTCGCCAGCTGGCGTAATAGCG
AAGAGGCCCGCACCGATCGCCCTTCCCAACAGTTGCGCAGCCTGAATGGCGAATGGCGCTTTGCCTGGTTTCCGGCACCAGAAGCGGTGCC
GGAAAGCTGGCTGGAGTGCGATCTTCCTGAGGCCGATACTGTCGTCGTCCCCTCAAACTGGCAGATGCACGGTTACGATGCGCCCATCTACA
CCAACGTGACCTATCCCATTACGGTCAATCCGCCGTTTGTTCCCACGGAGAATCCGACGGGTTGTTACTCGCTCACATTTAATGTTGATGAAAG
CTGGCTACAGGAAGGCCAGACGCGAATTATTTTTGATGGCGTTCCTATTGGTTAAAAAATGAGCTGATTTAACAAAAATTTAATGCGAATTTTAAC
AAAATATTAACGTTTACAATTTAAATATTTGCTTATACAATCTTCCTGTTTTTGGGGCTTTTCTGATTATCAACCGGGGTACATATGATTGACATGCTA
GTTTTACGATTACCGTTCATCGATTCTCTTGTTTGCTCCAGACTCTCAGGCAATGACCTGATAGCCTTTGTAGATCTCTCAAAAATAGCTACCCTC
TCCGGCATTAATTTATCAGCTAGAACGGTTGAATATCATATTGATGGTGATTTGACTGTCTCCGGCCTTTCTCACCCTTTTGAATCTTTACCTACAC
ATTACTCAGGCATTGCATTTAAAATATATGAGGGTTCTAAAAATTTTTATCCTTGCGTTGAAATAAAGGCTTCTCCCGCAAAAGTATTACAGGGTCA
TAATGTTTTTGGTACAACCGATTTAGCTTTATGCTCTGAGGCTTTATTGCTTAATTTTGCTAATTCTTTGCCTTGCCTGTATGATTTATTGGATGTT

Table S12: Imaging settings in microscopy experiments shown in main and supplemental figures.

Figure	571-630 nm detection		650-763 nm detection		STED PSF modulation	Pinhole / AU	Line accumulations	Pixel dwell time / µs	Pixel size / nm
	561 nm laser power / µW	775 nm laser power / mW	640 nm laser power / µW	775 nm laser power / mW					
1D, S1	5.7	240	4.7	240	2D	1.0	20	5	12,5
2, 3	3.7	157	3.2	109	2D	1.0	20-25	5	30
4, S5 (confocal)	3.3	-	3.2	-	-	1.0	5-10	5	50
4, S5 (STED)	3.3	109	3.2	91	2D	0.81	20-35	5	30
S6	1.5	45	3.2	28	3D (top hat)	0.71	7	17	60 (voxel size)

Table S13: Fraction of specific DNA origami signal analyzed from the total identified DNA Origami signal. Uncertainties are given as standard errors.

	1x	2x	3x	4x	5x	7x
Fraction of specific signal / %	92.1 ± 1.1	97.0 ± 1.4	94 ± 3	96.9 ± 1.7	96.3 ± 1.8	97 ± 3
